# Endothelial Dysfunction: Associations with Exposure to Ambient Fine Particles in Diabetic Individuals

**DOI:** 10.1289/ehp.11666

**Published:** 2008-07-31

**Authors:** Alexandra Schneider, Lucas Neas, Margaret C. Herbst, Martin Case, Ronald W. Williams, Wayne Cascio, Alan Hinderliter, Fernando Holguin, John B. Buse, Kathleen Dungan, Maya Styner, Annette Peters, Robert B. Devlin

**Affiliations:** 1 Helmholtz Zentrum München, German Research Center for Environmental Health, Institute of Epidemiology, Neuherberg, Germany; 2 Human Studies Division, National Health and Environmental Effects Research Laboratory, U.S. Environmental Protection Agency, Research Triangle Park, North Carolina, USA; 3 University of North Carolina, School of Medicine, Chapel Hill, North Carolina, USA; 4 Human Exposure and Atmospheric Sciences Division, National Exposure Research Laboratory, U.S. Environmental Protection Agency, Research Triangle Park, North Carolina, USA; 5 East Carolina School of Medicine, Greenville, North Carolina, USA; 6 Centers for Disease Control and Prevention, Atlanta, Georgia, USA; 7 Division of Endocrinology, Diabetes, and Metabolism, Ohio State University, Columbus, Ohio, USA; 8 Focus Network Nanoparticles and Health (NanoHealth), Helmholtz Zentrum München, German Research Center for Environmental Health, Neuherberg, Germany

**Keywords:** air pollution, diabetes, endothelial dysfunction, environmental epidemiology, particulate matter

## Abstract

**Background:**

Exposure to fine airborne particulate matter [≤2.5 μm in aerodynamic diameter (PM_2.5_)] has been associated with cardiovascular and hematologic effects, especially in older people with cardiovascular disease. Some epidemiologic studies suggest that adults with diabetes also may be a particularly susceptible population.

**Objectives:**

The purpose of this study was to analyze the short-term effects of ambient PM_2.5_ on markers of endothelial function in diabetic volunteers.

**Methods:**

We conducted a prospective panel study in 22 people with type 2 diabetes mellitus in Chapel Hill, North Carolina (USA), from November 2004 to December 2005. We acquired daily measurements of PM_2.5_ and meteorologic data at central monitoring sites. On 4 consecutive days, we measured endothelial function by brachial artery ultrasound in all participants and by pulsewave measurements in a subgroup. Data were analyzed using additive mixed models with a random participant effect and adjusted for season, day of the week, and meteorology.

**Results:**

Flow-mediated dilatation decreased in association with PM_2.5_ during the first 24 hr, whereas small-artery elasticity index decreased with a delay of 1 and 3 days. These PM_2.5_-associated decrements in endothelial function were greater among participants with a high body mass index, high glycosylated hemoglobin A1c, low adiponectin, or the null polymorphism of glutathione *S*-transferase M1. However, high levels of myeloperoxidase on the examination day led to strongest effects on endothelial dysfunction.

**Conclusions:**

These data demonstrate that PM_2.5_ exposure may cause immediate endothelial dysfunction. Clinical characteristics associated with insulin resistance were associated with enhanced effects of PM on endothelial function. In addition, participants with greater oxidative potential seem to be more susceptible.

Numerous epidemiologic studies have reported associations between exposure to ambient levels of particulate matter (PM) and various indices of acute cardiopulmonary morbidity and mortality [U.S. Environmental Protection Agency (EPA) 2004]. Ambient PM exposure at current levels has been implicated in the onset and exacerbation of lung and heart disease (U.S. EPA 2004). Although the primary mode of entry into the body is through the respiratory system, the greatest population-attributable risk from air pollution is due to cardiovascular disease.

Epidemiologic data suggest that individuals with diabetes may be at higher risk from effects of PM (O’Neill et al. 2007; Zanobetti and Schwartz 2001, 2002). Several parallels exist between the pathophysiologic effects of diabetes and the cardiovascular, hematologic, and autonomic responses to airborne PM. Moreover, evidence indicates that diabetes and insulin resistance are associated with endothelial dysfunction (Caballero 2003; Feener and King 2001; Hsueh and Quinones 2003; Schram et al. 2004), which suggests that people with diabetes may be particularly susceptible to the effects of PM.

Flow-mediated dilatation (FMD) of the brachial artery is a noninvasive method of assessing endothelial function that has been widely employed in clinical studies of vascular biology. The technique uses high-definition ultrasound to measure brachial artery diameter before and after an increase in shear stress that is induced by reactive hyperemia. The arterial dilatation, quantified as the percent change in arterial diameter, reflects local endothelial release of nitric oxide (NO). This endothelial-dependent response to increased shear stress can be contrasted to the endothelium-independent dilatation observed with nitroglycerin. As an index of endothelial function, FMD can be viewed as a “barometer” of vascular health and is considered a reasonable surrogate marker for assessing atherosclerosis (Perticone et al. 2001; Sorensen et al. 1997).

Endothelial dysfunction plays a significant role in the atherosclerotic process (Bonetti et al. 2003; Libby et al. 2002). Impaired function of the vascular endothelium is associated with a number of vascular changes, such as decreased vasodilatation, development of prothrombotic and proinflammatory states, and smooth muscle cell proliferation, all of which contribute to the formation and progression of chronic atherosclerotic lesions (Widlansky et al. 2003). An increased incidence of adverse cardiovascular events has been reported in subjects with impaired endothelial function compared with subjects with preserved endothelial function (Gokce et al. 2002, 2003; Heitzer et al. 2001; Perticone et al. 2001). Therefore, endothelial dysfunction as a reaction to increased ambient PM might provide further mechanistic insight into the observed associations between PM exposure and increased morbidity and mortality. Several previous studies have suggested that exposure to air pollutants results in endothelial dysfunction (Briet et al. 2007; Brook et al. 2002; O’Neill et al. 2005) in healthy volunteers.

We conducted this study to examine the effects of fluctuations in ambient PM exposure, measured at a local air monitoring station, on changes in endothelial function parameters among adults with type 2 diabetes. We hypothesized that exposure to increased ambient air pollutants results in further impairment of endothelial function. Moreover, we analyzed effect modification by clinical characteristics associated with insulin resistance such as body mass index (BMI), glycosylated hemoglobin A1c (HbA1c), and adiponectin and by daily myeloperoxidase (MPO) level. MPO is an enzyme that binds to the vessel wall, critically modulates structural and humoral integrity of the vessel wall, and depletes vascular NO bioavailability (Eiserich et al. 2002; Nicholls and Hazen 2005; Rudolph et al. 2006).

To identify susceptible subgroups, we also analyzed effect modification by genes of the antioxidant defense family and the hemochromatosis gene (*HFE*). The protein product of the *HFE* gene modulates iron binding and storage (Hanson et al. 2001) from pulmonary sources. The analyzed polymorphisms are associated with increased iron uptake (Feder et al. 1998) and may modify the toxic effect of metal-rich PM on the cardiovascular system because iron stores are inversely associated with the gastrointestinal absorption of potentially toxic metals (Flanagan et al. 1980).

## Materials and Methods

### Study population

We identified 22 volunteers, 48–80 years of age, with type 2 diabetes through the University of North Carolina–Chapel Hill (UNC) Diabetes and General Medicine Clinics and through newspaper advertisements. Potential participants had to meet the following inclusion criteria: *a*) a diagnosis of type 2 diabetes, but without taking insulin; *b*) a stable medication regimen throughout their participation; and *c*) an electrocardiogram demonstrating normal sinus rhythm. We excluded potential participants for any of the following criteria: *a*) a current smoker or a smoking history, defined as more than one pack of cigarettes within the year before enrollment; *b*) a hematocrit < 36%; *c*) a medical history or health problems that preclude participation, as decided by the study physician, such as presence of a pacemaker or implanted cardioverter defibrillator, history of atrial fibrillation, history of solid organ transplant, dialysis therapy, active cancer or history of cancer within the last 5 years, hepatitis B or C, unstable angina, hypersensitivity to nitroglycerin/nitrates/nitrites, or respiratory tract infection within the preceding 4 weeks; *d*) a recent vascular event or intervention (< 6 months or < 1 year ago, depending on the type of intervention), such as coronary artery graft bypass surgery or percutaneous coronary intervention; or *e*) pregnancy. Participants were asked to refrain from vigorous exercise on study mornings and to refrain from taking antioxidants (e.g., vitamins C and E), fish oil, niacin, arginine, over-the-counter vasoactive agents (e.g., decongestants), and anti-inflammatory agents (e.g., ibuprofen, naproxen, aspirin) unless it was prescribed as a daily medication (in which case it was continued), for the week before the study as well as the week of the study. Participants were also asked to refrain from use of phosphodiesterase enzyme inhibitors during the week of the study.

Each participant visited the U.S. EPA Human Studies Facility (HSF) in Chapel Hill, North Carolina, on 5 consecutive weekdays between November 2004 and December 2005. We obtained data on health status, pulmonary and cardiac symptoms, medication, and smoking history at baseline and during four follow-up visits. Altogether, a maximum of 88 observations were available for analysis, depending on the examined health outcome. All volunteers signed a written consent form, and the study protocol was approved by the UNC Human Studies Biomedical Institutional Review Board and by the U.S. EPA.

### Clinical measurements

#### Clinical procedures

On Monday morning of each examination week, the participants filled out a baseline questionnaire. In each of the next four mornings, participants checked into the medical station under fasting conditions and without having taken their antidiabetic medication. Upon first arriving at the medical station each morning, a glucometer was used to evaluate fasting glucose levels.

A blood sample was obtained from each participant at each visit and analyzed by a clinical laboratory (Lab Corp., Burlington, NC, USA) for a full blood panel, including adiponectin, homocysteine, and HbA1c. MPO was detected using a Human Cardiovascular Disease Panel (Lincoplex, Linco Research, Inc., St. Charles, MO, USA) run on a Luminex 100 Multiplex system. MPO, homocysteine, and adiponectin were measured during each visit, whereas HbA1c was measured only once. In addition, DNA analysis was performed with regard for the null polymorphism of glutathione *S*-transferase M1 (*GSTM1*) (chromosomal location 1p13.3), the valine-coding polymorphism (Ile105Val) of *GSTP1* (chromosomal location 11q13), the aspartic acid-coding polymorphism H63D (His63Asp) of the *HFE* gene (chromosomal location 6p21.3), the tyrosine-coding polymorphism C282Y (Cys282Tyr) of the *HFE* gene, and the serine-coding polymorphism P149S (Pro149Ser) of the quinone oxidoreductase (*NQ01*) gene (chromosomal location 16q22.1).

Participants were then escorted to the UNC General Clinical Research Center, where vascular reactivity was assessed by measuring flow-mediated and nitroglycerin-mediated dilatation (NTGMD) of the brachial artery via ultrasound. In 13 participants, pulse waveform measurements were performed to obtain a measure of arterial stiffness. Soon after the measurements were completed, participants took their antidiabetic medication together with a diabetic snack.

#### Endothelial function and vascular compliance measurements

We measured FMD and NTGMD of the brachial artery using an HDI 5000 ATL ultrasound machine equipped with a 12.5-MHz transducer (Philips, Bothell, WA, USA) according to the guidelines published by Corretti et al. (2002). A resting blood pressure was measured at baseline. Images of the right brachial artery were then acquired at rest and during reactive hyperemia for quantification of FMD. After baseline images were recorded, hyperemia was induced by inflating a pneumatic tourniquet proximal to the brachial artery to 50 mmHg above systolic pressure for 5 min. Images of the brachial artery were acquired for 90 sec after abrupt cuff deflation. NTGMD was then assessed. A second baseline image was acquired after 15 min of rest, and a final image was then recorded 5 min after administration of 400 μg sublingual nitroglycerin spray.

Gated end-diastolic images of the brachial artery were stored in a digital format for subsequent analysis. We measured arterial diameter from the lumen–intimal interfaces of the proximal and distal walls using customized software (Brachial Tools, Medical Imaging Applications, LLC, Coralville, IA, USA). Data from at least three consecutive end-diastolic frames were averaged for each baseline measurement, and from at least three frames at maximal dilatation during reactive hyperemia and after administration of nitroglycerin. FMD and NTGMD were calculated as the percentage change in vessel diameter from their respective baselines. FMD is a measure of endothelial-dependent vasomotion and is due largely to endothelial release of NO, whereas the endothelium-independent dilatation observed in response to nitroglycerin reflects vascular reactivity to an exogenous NO donor.

We assessed vascular compliance (arterial elasticity) by the contour analysis of the arterial pressure waveform (pulsewave) using the HDI/PulseWave CR-2000 Research Cardiovascular Profiling System (Hypertension Diagnostics, Inc., Eagan, MN, USA). According to the method described by Cohn et al. (1995), a blood-pressure cuff was placed around the participant’s left upper arm and a stabilizer was placed on the participant’s right wrist for optimal positioning and minimal movement during data collection. After palpating the strongest radial pulsation, we placed a piezoelectric sensor perpendicular to the skin overlying the right radial artery to capture an analog blood pressure waveform data signal. The tonometer sensor array adjusts automatically until it obtains stable waveform. We then obtained three assessments of arterial compliance and averaged them for analyses. The system determined capacitive compliance of the proximal aorta and major branches [large-artery elasticity index (LAEI)], sinusoidal oscillatory compliance of the distal arteries [small-artery elasticity index (SAEI)], and systemic vascular resistance (SVR), all indirect measures of endothelial function. Endothelial dysfunction can be associated with decreasing elasticity indices and an increase in SVR. Using a noninvasive, direct acoustic transducer, the system gathered and analyzed a 30-sec analog tracing of the radial artery waveforms digitized at 200 samples/sec. A beat determination was made during that 30-sec period determining systole, peak systole, onset of diastole, and end of diastole. Representative averaged waveforms of individual beats were analyzed using a parameter-estimating algorithm (Finkelstein and Cohn 1992) to fit a multiexponential model based on the Windkessel model for identifying the three pulse waveform indices (O’Rourke and Gallagher 1996).

### Air pollution monitoring

We obtained daily 24-hr concentrations (midnight to midnight) of official ambient PM_2.5_ (PM ≤ 2.5 μm in aerodynamic diameter) mass network data from a monitoring station located approximately 44 km (27 miles) east of the HSF. In addition, we measured concentrations of ambient PM_2.5_ mass (0900–0900 hours) with a 3000K Versatile Air Pollution Sampler (URG Corp., Chapel Hill, NC, USA) (Williams et al. 2000) located on the HSF rooftop approximately 30 m above ground level. The Spearman correlation between both PM_2.5_ measurements was 0.85, and the rooftop data was used to impute 3 days of missing network data based on a linear regression model.

We obtained continuous 2-min measurements of air temperature, barometric pressure, and relative humidity from the HSF rooftop and calculated 24-hr averages if 240 (33%) or fewer 2-min values were missing on any day. The rooftop data were complete, so no imputation was necessary.

### Statistical analyses

The study was conducted as a panel study with four repeated measurements per participant. Thus, every person acted as his or her own control, which limited the need for an adjustment for patient characteristics in the analysis.

We analyzed data using the SAS, version 9.1, statistical package (SAS Institute, Inc., Cary, NC, USA). For the analysis of the PM effect, we used additive mixed models with a random participant effect and “compound symmetry” covariance structure (Greven et al. 2006). We built models to identify meteorologic and temporal determinants as potential confounders, for each outcome variable separately. We assessed model fit by the Akaike Information Criterion (AIC). In a first selection step, we considered air temperature, relative humidity, and barometric pressure as potential confounders of the association between health parameters and network PM_2.5_. For all three meteorologic parameters, we checked individual lags at lag 0 to lag 4 and the 5-day moving-average as possible determinants—either linear or as penalized smoothing splines with a maximum of 20 knots. After the selection of meteorology, we examined day of week and season as determinants. We defined season as “cold” from 19 November 2004 to 31 March 2005 and from 1 November 2005 to 9 December 2005, and as “warm” from 1 April 2005 to 31 October 2005. None of the above determinants were selected for the model if the AIC showed no improvement in model fit.

We considered the exposure, in this case ambient PM_2.5_ mass, as an immediate (lag 0), a delayed (lag 1 to lag 4), or a cumulative linear effect over 5 days. We present effect estimates as percent changes of the mean outcome variable together with 95% confidence intervals (CIs) for a 10-μg/m^3^ increment in ambient PM_2.5_ mass.

We analyzed sensitivity by changing the covariance structure to first-order autocorrelation. Moreover, for significant results, we applied a model with random slopes for the PM effect to assess the individual responses of the participants.

We performed effect modification analyses using dichotomous indicator variables and an interaction term. We considered the influence of BMI (cut point, 30 kg/m^2^), HbA1c (cut point, 7%), adiponectin (cut point, 3,700 ng/mL), homocysteine (cut point, 12 μmol/L), MPO (cut point, 7 ng/mL), and statin intake (yes/no). Cut points were typically near the median value seen in the 22 participants. In addition, we assessed gene–environment interaction analyses for the polymorphisms of *GSTM1*, *GSTP1*, *HFE* H63D, *HFE* C282Y, and *NQ01* P149S. Because we assessed only 13 participants for pulse waveform response, the sample size was too small to analyze the potential effect modification of *GSTP1*, *HFE*, or *NQ01*.

## Results

### Participant characteristics

The participants were 48–78 years of age, and their BMIs were 20–44 kg/m^2^. Almost two-thirds of the recruited volunteers were male (Table 1). All participants were current nonsmokers, but almost half of them were ex-smokers. About half of the participants were obese, and < 50% had a systolic blood pressure > 140 mmHg, indicating mild hypertension. Duration of type 2 diabetes ranged from 2 months to 23 years (Table 2). Most participants had a history of hypertension and dyslipidemia. Almost one-third of the participants had a combination of elevated HbA1c, elevated homocysteine, and low adiponectin values.

Most participants were being treated with oral antihyperglycemic medications such as metformin, sulfanylureas, and thiazolidinediones, and half of them were taking statins and antihypertensives. About two-thirds of the participants regularly took aspirin.

### Description of vascular measures

The average values of the vascular measures were typical, with a 5.9% increase in brachial artery diameter after reactive hyperemia and a 13.4% increase in diameter after administration of nitroglycerin (Table 3). Responses to reactive hyperemia and nitroglycerin were independent, showing a median within-patient Spearman correlation of 0.09. Of the 13 volunteers with additional pulse waveform measurements, one had to be excluded as an overly influential outlier for the SAEI analysis because he showed extremely high values compared with the range of all other observations. We therefore analyzed the association for SAEI and PM_2.5_ only among the remaining 12 participants. Correlations between FMD and the three pulse waveform measurements LAEI, SAEI, and SVR were very weak, with correlation coefficients of 0.19, −0.17, and 0.36, respectively.

### Air pollutant and meteorology measurements

**Figure 1:**
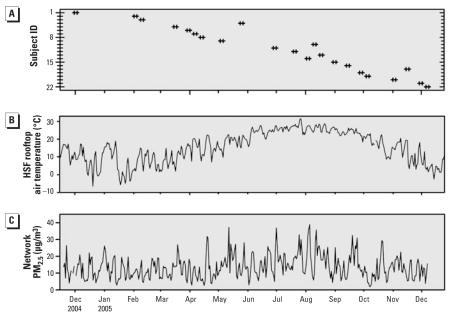
Patient visits, air temperature, and PM_2.5_ between 19 November 2004 and 9 December 2005. (*A*) Data points represent the week each of the 22 participants visited the HSF. (*B*) Daily 24-hr temperature averages (midnight to midnight) calculated from continuous 2-min data collected from the HSF rooftop. (*C*) Imputed 24-hr average PM_2.5_ concentrations (midnight to midnight) collected at the official network station.

The 24-hr PM_2.5_ values over the study period were generally below the U.S. National Ambient Air Quality Standard (NAAQS) of 35 μg/m^3^ (U.S. EPA 2006), and the mean over the study period was less than the annual average NAAQS of 15 μg/m^3^ (Table 3). Participant visits were fairly uniformly distributed over the study period (Figure 1A). Air temperature (Figure 1B) showed the expected seasonal variation, whereas the PM_2.5_ concentrations (Figure 1C) showed mostly daily variations with little seasonal pattern. Median within-patient Spearman correlation can be found in the Supplemental Material, Table 1 (http://www.ehponline.org/members/2008/11666/suppl.pdf).

### Association between air pollution and vascular parameters

FMD was significantly associated with PM_2.5_ levels on the same day (lag 0), suggesting that endothelial dysfunction is a relatively rapid consequence of exposure to PM (Table 4). The estimates given in Table 4 correspond to an absolute mean change in FMD (lag 0) per 10-μg/m^3^ increase in PM_2.5_ of −1.0% (95% CI, −2.0 to 0.0). There was no significant association of NTGMD with PM_2.5_ on the same day, suggesting that the decrease in FMD with increased PM_2.5_ is endothelium dependent and does not reflect increased smooth muscle cell reactivity. There was, however, a decrease in NTGMD with a lag of 1 day, suggesting the possibility of an impairment of smooth muscle cell responsivity to nitroglycerin with a slight delay. SAEI decreased significantly with a lag of 1 and 3 days and also with the 5-day average, whereas SVR increased after 2 and 4 days. Similar to SAEI, LAEI decreased in association with PM_2.5_ with a lag of 3 days (Table 4).

The participant-specific changes in FMD and SAEI were quite homogeneous for all the participants (*p*-values for heterogeneity were not significant), suggesting that the response was not driven by a small subset of individuals [see Supplemental Material, Figure 1 (http://www.ehponline.org/members/2008/11666/suppl.pdf)]. The significant association of PM and SAEI at lag 3 was sensitive to the exclusion of the influential outlier.

The estimated effects were not sensitive to changing the covariance matrix from compound symmetry to first-order autocorrelation.

### Effect modification analysis

**Figure 2:**
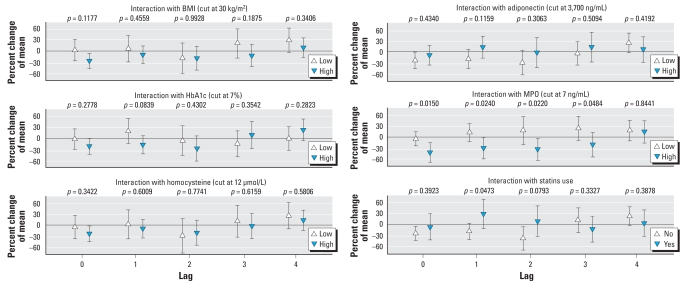
Effect modification of FMD by markers representing diabetic disease control or medication use. The estimates are given as percent change of mean FMD level based on a 10-μg/m^3^ increment of PM_2.5_; error bars indicate 95% CIs. *p*-Values indicate the significance of the interaction term.

Diabetes is a heterogeneous disorder with regard to disease severity, disease control, and medication use. Among the diabetic participants, PM_2.5_-associated changes in FMD were modified by several of such clinical characteristics associated with insulin resistance (Figure 2). Participants with high BMI tended to have a greater response to PM_2.5_. Individuals with, on average, high HbA1c, low plasma adiponectin, or high homocysteine and participants not treated with statins also showed increased responsiveness. However, interaction terms were statistically significant only for MPO. Individuals with elevated MPO levels on the examination day had greater impairment of FMD in association with PM_2.5_ than did individuals with low MPO levels on that day. Interestingly, in the subgroup with high MPO levels, the significant decrease in FMD remained also for lag 1 and lag 2.

**Figure 3:**
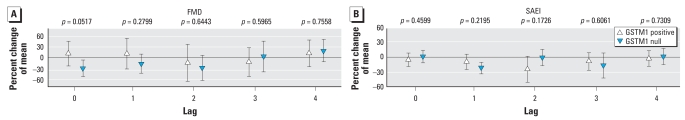
Effect modification of FMD (*A*) and SAEI (*B*) by the *GSTM1* genotype. The estimates are given as percent change of mean FMD level based on a 10-μg/m^3^ increment of PM_2.5_; error bars indicate 95% CIs. *p*-Values indicate the significance of the interaction term.

With regard to gene–environment interaction analysis, we detected a clear effect modification by the *GSTM1* null polymorphism (deletion on both alleles) for FMD (lag 0). We found a similar association for SAEI with lag 1 (Figure 3). For *GSTP1*, we examined the wild type and a variant in which isoleucine is replaced by valine. We assessed dominant, recessive, and additive models to examine *GSTP1* gene–environment interactions, and an additive model proved to be the best choice. With each copy of the valine-coding allele for *GSTP1*, the PM_2.5_-associated decrement in FMD (lag 0) showed an interesting, but not statistically significant, trend: −12.5% (95% CI, −45.0 to 20.1) for the wild type, −18.2% (95% CI, −37.6 to 1.2) for one copy of the valine-coding allele, and −23.9% (95% CI, −56.3 to 8.4) for two copies.

The interaction with the *HFE* polymorphism *HFE* H63D (wild-type coding for his-tidine to variant coding for aspartic acid) could be analyzed only with a dominant model because only 4 of the 22 participants had this variant, and none of them was homozygous. The participants with wild type showed a PM_2.5_–associated decrement (not statistically significant) in FMD (lag 0) of −15.0% (95% CI, −37.7 to 7.7), whereas the four participants with one copy of the variant showed −30.1% (95% CI, −70.9 to 10.7).

Because of small sample numbers, we did not observe statistically significant interactions with other analyzed gene polymorphisms (*HFE* C282Y and *NQ01* P149S).

## Discussion

We analyzed markers of endothelial function in diabetes type 2 participants in association with day-to-day changes of PM_2.5_ in a repeated measurement framework. We found FMD of the brachial artery to be decreased on the same day with increasing ambient PM_2.5_ exposure.

### Endothelial function in general

The endothelium is composed of cells that not only serve as a physical barrier between blood and tissues but also act to maintain vascular homeostasis, the dynamic balance between vasodilatation and vasoconstriction, by synthesizing and releasing substances that modulate vascular tone and structure. In addition, it plays an important role in the interaction of circulating cells with the vessel wall. It inhibits and stimulates smooth muscle cell proliferation and migration and takes part in processes of thrombosis and fibrinolysis (Cohen 2007; Mahmoudi et al. 2007). Endothelial dysfunction is uniformly recognized to be characterized by loss of normal endothelium-dependent vasodilatation (Mahmoudi et al. 2007). Impaired endothelial function is associated with nearly all known cardiovascular risk factors (Celermajer et al. 1994; Ganz and Vita 2003; Libby et al. 2002; Ross 1999; Vita and Keaney 2002) and is predictive of future cardiovascular events (Gokce et al. 2002, 2003; Heitzer et al. 2001). It can be used to identify individuals at risk before the development of clinically apparent cardiovascular disease. FMD, as an index of endothelial function, represents an excellent measure of underlying vascular health and reflects the combined influence of the known and unknown processes that contribute to atherosclerosis (Kardasz and De Caterina 2007; Vita and Keaney 2002; Widlansky et al. 2003). Because endothelial dysfunction plays a central role in the pathogenesis of cardiovascular disease, it may underpin the link between air pollution and the risk of an acute coronary event.

Endothelial cells mediate vasodilatation predominantly through the production of NO. NO diffuses into the underlying smooth muscle cells, where it is responsible for smooth muscle cell relaxation and vascular dilatation. NO produced by endothelial cells also has anti-inflammatory effects, which include a decrease in soluble vascular cell and intercellular adhesion molecules-1, E-selectin, and tumor necrosis factor α(Hsueh et al. 2004).

### Endothelial function in diabetes

Type 2 diabetes is characterized by insulin resistance. The parallel progression between insulin resistance and endothelial dysfunction suggests a close relationship between the two (Hsueh et al. 2004). Insulin stimulates NO production from endothelial cells, so people with diabetes usually have lower baseline levels of NO than do subjects without diabetes (Calles-Escandon and Cipolla 2001; Creager et al. 2003; Quyyumi 1998).

### Diabetes and PM

People with diabetes have been shown to be at greater risk for morbidity and mortality associated with exposure to PM (Goldberg et al. 2006; Zanobetti and Schwartz 2002). Although many people with diabetes have secondary cardiovascular complications, as a group they show larger effect sizes to PM than do people with cardiovascular disease and no diabetes (Zanobetti and Schwartz 2001). One possibility for the added responsiveness may be that people with diabetes have endothelial dysfunction. If vascular endothelial cells in these individuals are compromised, they may be more susceptible to the effects of PM. Additionally, people with diabetes are known to have disproportional reactive oxygen species formation, which has been associated with increased tissue damage (Maritim et al. 2003) and enhanced smooth muscle tone (Stocker and Keaney 2004). They also have endothelial inflammation and hypercoagulability (Beckman et al. 2002).

PM has been shown to cause adverse health effects through many of these same mechanisms. The exposure to ambient PM might lead to pulmonary as well as systemic inflammation (Pope and Dockery 2006). Moreover, PM or PM components have been found to be translocated from the lung into the vascular system (Nemmar et al. 2002, 2004). Thus, diabetes and the vascular effects of PM may share common pathways and interact to enhance responsiveness of diabetic patients to air pollutants. Of note, PM has been shown to induce oxidative stress (Donaldson et al. 2005), and this excessive generation of free radicals may also affect the antioxidant capacity in the endothelium and lead to further synergistic interaction on the generation of reactive oxygen species after PM exposure. However, this argument must be approached with the caveat that PM_2.5_ probably does not adequately represent the toxicity of ultrafine PM to the circulatory system.

### Endothelial function and PM

Other studies have also examined the association of PM and endothelial cell function. Inhalation of diesel exhaust has been shown to impair vasomotor responses to endothelium-dependent and -independent vasodilators 6 hr after exposure (Mills et al. 2005). In a French study (Briet et al. 2007) with 40 young healthy male volunteers devoid of cardiovascular risk factors, exposure to gaseous and PM pollutants led to a significant alteration in endothelial function in large and small arteries, respectively. Brook et al. (2002) found a significantly decreased basal brachial artery diameter in individuals exposed to concentrated ambient air PM plus ozone for 2 hr. However, in contrast to our findings, they observed no altered FMD in those individuals. This difference might either be explained by the study population (normal vs. diabetes), duration of exposure (2 hr vs. 24 hr), or the presence of ozone in the study of Brook et al. Healthy intercollegiate athletes showed decreased FMD when exposed to ambient PM while exercising (Rundell et al. 2007). O’Neill et al. (2005) studied 270 Greater Boston, Massachusetts, residents, including 182 individuals with type 2 diabetes. In this subgroup, they found the strongest decrease of FMD associated with a 6-day moving-average exposure of sulfates and black carbon; the effect estimates for PM_2.5_ and particle number count were a little smaller and not statistically significant. In addition, several studies on passive smoking reported a decrease in endothelial function after exposure to environmental tobacco smoke (Holay et al. 2004; Howard and Wagenknecht 1999; Kato et al. 2006).

Impaired endothelium-dependent vasodilatation could be caused by decreased production of NO by endothelial cells or by PM-induced changes in smooth muscle cells lining the vessel. To distinguish between these two possibilities, participants in our study were given nitroglycerin, an NO donor, as part of the vascular imaging procedure. There was no significant association at lag 0 between PM and NTGMD, suggesting that the change in FMD at lag 0 is best explained by an alteration in endothelial cell NO production rather than some other effect in the vascular smooth muscle cells. Several studies have shown an increased risk for cardiovascular events in people in whom endothelial cell production of NO is chronically reduced. There are also reports that a sudden loss in endothelial activity could promote cardiac ischemia or trigger instability of susceptible plaques in individuals with flow-limiting obstructive lesions (Muller et al. 1994). An alternative putative measure of endothelial function in a subsample of our study showed increased systemic arterial stiffness with increasing PM_2.5_. Reductions in LAEI increase with age, whereas reductions in SAEI are observed in patients at risk for coronary heart disease and independently predict risk for cardiovascular events (Grey et al. 2003). Stiffer vessels produce an earlier return of the reflected wave from vascular branching points to the ascending aorta. This phenomenon increases left ventricular afterload on the heart and leads to reduced coronary perfusion (Weber et al. 2004). Endothelial dysfunction and increased arterial stiffness commonly coexist in patients at increased risk of cardiovascular disease such as those with diabetes (De Vriese et al. 2000). This leads to the hypothesis that cardiovascular risk factors may exert their detrimental effects on arterial stiffness through endothelial dysfunction and that endothelial factors such as NO may contribute a functional component to arterial stiffness. Further studies are required to fully define the link between endothelial function and arterial stiffness. However, most studies suggest that endothelium dysfunction might be one potential mechanism underlying alterations on the elasticity of atherosclerotic vessels, supporting our findings about immediate changes in endothelial function (FMD) and delayed changes in arterial stiffness (pulse waveform measures) in association with increasing PM.

### Effect modification

In our study, markers of insulin resistance such as obesity and poor glycemic control as reflected by increased HbA1c and adiponectin were associated with response to PM_2.5_. Adiponectin is an abundant circulating adipocyte-derived peptide, has insulin-sensitizing, anti-inflammatory, and cardioprotective properties (including endothelial function), and is inversely correlated with BMI (Tan et al. 2004). Low adiponectin levels are associated with an increased risk of developing type 2 diabetes in healthy individuals (Spranger et al. 2003).

The observed effect modification is in agreement with O’Neill et al. (2007), who demonstrated that elevated BMI in diabetics was also associated with increased responsiveness to PM. However, we found the strongest effect modification for MPO, an enzyme that binds to the vessel wall and depletes vascular NO bioavailability (Rudolph et al. 2006). Activated polymorphonuclear neutrophils, an early event in unstable coronary disease, liberate MPO, which has oxidative power. Therefore, it is conceivable that on days with elevated PM levels, there is increased MPO production, resulting in less NO production from endothelial cells and thus a decrease in endothelial function.

We also observed that participants with the null genotype for *GSTM1* had larger decrements in FMD associated with PM exposure. *GSTM1* is a phase II enzyme that can scavenge oxygen free radicals, metabolize reactive oxygen species, and detoxify xenobiotics present in PM. Therefore, it is plausible that people with a *GSTM1* deletion are not able to handle oxidative stress well and may be more responsive to agents such as PM that increase oxidative stress. Asthmatic children with the *GSTM1* null genotype appear to be more susceptible when exposed to ozone than are those with the wild-type allele (Romieu et al. 2004, 2006). Gilliland et al. (2004) showed that in the presence of diesel exhaust particles, the enhancement of allergic responses was largest in participants with *GSTM1* null genotype. Moreover, Schwartz et al. (2005) reported a stronger association between autonomic control of the heart and PM in people with the null *GSTM1* allele.

The trends found for *GSTP1*, also a phase II enzyme and a crucial factor in determining the sensitivity of cells to toxic chemicals and products of oxidative stress, point in the same direction as the *GSTM1* results. The *GSTP1* gene product provides > 90% of the *GST* family activity in the lung; enhancement of the allergic response by secondhand tobacco smoke was larger in allergen-sensitive individuals with the *GSTP1* wild type (Gilliland et al. 2006). However, in the present study, an additive trend of the variant showed more impaired FMD with every copy of the variant. Salam et al. (2007) similarly found that the variant contributed to the occurrence of childhood asthma and increase of asthma susceptibility in association with exposures to major roads. Also Gilliland et al. (2002) showed slower lung function growth in children who were homozygous for the variant.

Polymorphisms of the *HFE* gene have been found to blunt the effect of particles on cardiac autonomic function in the Normative Aging Study. Park et al. (2006) observed PM_2.5_ effects only in individuals with the wild-type of the HFE genotype; a single copy of the variant blunted the relationship. The protein product of the *HFE* gene modulates iron binding and storage (Hanson et al. 2001) from pulmonary sources. The two analyzed polymorphisms are associated with increased iron uptake (Feder et al. 1998) and may modify the effect of metal-rich particles on the cardiovascular system. However, in contrast to results of Feder et al. (1998), we found that diabetics with one copy of the variant (no one had two copies of the variant) had a trend toward stronger impairment of endothelial reactivity than did patients with the wild type.

### Strengths and limitations

One strength of the present study is the good compliance of the volunteers who all came four times for a clinical visit. However, the design might not be perfect for markers with longer half-lives, such as fibrinogen, because it implies autocorrelation between the data from consecutive days. This should not be a major influence on markers with shorter half-lives, such as measures of endothelial function. Daily variations could have an influence on the health outcomes, but models were adjusted for day of week if AIC proved it to be necessary.

A limitation of the study is the small number of subjects studied. Although sufficient to observe associations between PM and endothelial cell function in the whole population, power was too low to detect changes in subsets such as seen with some of the genetic polymorphisms.

Moreover, we have to acknowledge the possibility that the reported effect estimates might be modified by copollutants that were not measured. Because of space limitations, data on subject-specific PM monitoring and particle components in association with endothelial function will be presented in a future publication.

Another strength of this study is the investigation of air pollution effects in a particularly vulnerable subgroup. This population is thought to be especially vulnerable to the acute effects of particles due to chronic inflammation, imbalances in vasoactive processes, vascular remodeling, and an increased potential for oxidative stress. On the other hand, the results therefore cannot be generalized to the whole population. Of course, who is susceptible depends on the specific end point evaluated and the level and length of exposure. Although the results might not be generalizable to all diabetics, we believe they are generalizable to the vast majority, including those at risk for cardiac events.

Comparing two different aspects of endothelial function is certainly a strength of this study, especially examining the correlation between the two measurements and the lag structure in association with PM response. The use of pulse waveform analysis may therefore help to estimate the association between PM_2.5_ exposure with NO-independent pathways (not related to FMD, e.g., the one mediated by endothelin) compared with NO-mediated mechanisms of vascular injury (related to FMD). This aspect is important because animal data support that particle-induced vasoconstriction is mediated by endothelin release (Bouthillier et al. 1998). Moreover, the comparison of changes in FMD and NTGMD gave more insight into the pathophysiologic process than do existing studies on PM effects on endothelial dysfunction.

In this study, we used daily PM_2.5_ mass values from a state monitoring station located 27 miles away from the U.S. EPA HSF. In addition, we obtained PM_2.5_ values on the HSF rooftop. There was a strong correlation between these two sets of measurements, suggesting that we were accurately assessing ambient exposure of the study participants. However, our study shows the general limitation of accurately measuring exposure of participants as all panel studies with a similar design. The design and the limited number of participant also made it difficult to test for seasonal differences in the detected associations. All the subjects lived within a 30-mile radius of Chapel Hill, and no one had any unusual exposure to air pollution because of residence or occupation. Of course, there could be variable exposure to traffic as the participants commuted to work and to the U.S. EPA HSF, which is difficult to capture.

## Conclusions

Our study showed an immediate association between altered endothelial function and PM in persons with type 2 diabetes. Markers for insulin resistance (BMI, HbA1c, adiponectin) were associated with enhanced effects of PM on endothelial function. Individuals with greater oxidative potential were more susceptible to PM exposure. These data suggest that the adverse cardiovascular consequences of air pollution in patients with diabetes may be mediated, at least in part, by impaired function of the vascular endothelium, and that people with diabetes are an especially sensitive subpopulation that needs to be protected from the harmful effects of air pollution. The role of airborne PM in the pathophysiology of insulin resistance, diabetes, and their complications requires further study but does suggest opportunities to promote better health.

## Figures and Tables

**Table 1:** **Description of the study population characteristics: current nonsmoking subjects with type 2 diabetes mellitus.**

Characteristic	All subjects (*n* = 22)	Pulse waveform subjects (*n* = 13)
Age (years)	61 ± 8	59 ± 6
Male sex	14 (64)	7 (54)
Ethnicity		
Caucasian	15 (68)	7 (54)
African American	6 (27)	6 (46)
Hispanic American	1 (5)	0 (0)
BMI (kg/m^2^)	33 ± 7	34 ± 7
≥ 30 kg/m^2^	12 (55)	7 (54)
Average systolic blood pressure ≥140 mmHg	9 (41)	6 (46)
Smoking		
Never-smoker	12 (55)	8 (62)
Ex-smoker	10 (45)	5 (38)
Null polymorphism of *GSTM1*	10 (45)^a^	5 (38)^b^
Valine-coding polymorphism of *GSTP1*	10 (45)^c^	—d
Aspartic acid–coding polymorphism H63D of *HFE* gene	4 (18)^e^	—d
Tyrosine-coding polymorphism C282Y of *HFE* gene	1 (5)^f^	—d
Serine-coding polymorphism P149S of *NQ01* gene	4 (18)^g^	—d

Values shown are mean ± SD or no. (%).

a Four subjects declined permission for genetic testing.

b Two subjects declined permission for genetic testing.

c Two (9%) subjects were homozygous for the minor allele.

d Sample size was too small for stratification on genotype.

e No subjects were homozygous for the minor allele.

f No subjects were homozygous for the minor allele.

g One (5%) subject was homozygous for the minor allele.

**Table 2:** **Description of the study population clinical characteristics: current nonsmoking subjects with type 2 diabetes mellitus.**

Characteristic	All subjects (*n* = 22)	Pulse waveform subjects (*n* = 13)
Disease history		
Type 2 diabetes mellitus	22 (100)	13 (100)
Time since diabetes diagnosis (years)	6.4 ± 5.0	6.3 ± 6.0
Hyperlipidemia	19 (86)	11 (85)
Hypertension	19 (86)	11 (85)
Past myocardial infarction	0 (0)	0 (0)
Coronary artery disease	4 (18)	1 (8)
Peripheral vascular disease	3 (14)	1 (8)
Cerebrovascular disease	1 (5)	0 (0)
Diabetic retinopathy	1 (5)	0 (0)
Diabetic nephropathy^a^	8 (36)	7 (54)
Blood marker levels		
HbA1c^b^	6.7 ± 0.9	
≥ 7%^b^	9 (41)^c^	6 (46)
Homocysteine^b^	12.9 ± 3.4	
≥12 μmol/L^b^	11 (50)^c^	7 (54)^d^
Adiponectin^b^	4,727 ± 4,067	
< 3,700 ng/mL^b^	11 (50)^d^	7 (54)
MPO^b^	9.8 ± 7.3	
≥7 ng/mL^b^	41 (51)	24 (47)
Medication use		
Sulfonylureas	10 (45)	6 (46)
Thiazolidinediones	6 (27)	2 (15)
Metformin	14 (64)	10 (77)
Statins	12 (55)	7 (54)
Aspirin	14 (64)	9 (69)
Beta-blockers	9 (41)	4 (31)
Angiotensin-converting enzyme inhibitors	12 (55)	8 (62)
Calcium blockers	2 (9)	1 (8)
Diuretics	8 (36)	6 (46)
Angiotension II receptor blocker	3 (14)	2 (15)
Estrogen	2 (9)	2 (15)

Values shown are mean ± SD or no. (%).

a Based on the screening urine ( > 30 μg albumin/mg creatinine) on spot collection.

b HbA1c was measured only once, but homocysteine, adiponectin, and MPO were measured on all four visits. For the interaction analysis, we used the mean of the four measurements per subject for homocysteine and adiponectin. For MPO, we used all observations per patient because it showed high daily variation within subjects.

c Data were missing from two subjects.

d Data were missing from one subject.

**Table 3:** **Description of health parameters, PM2.5, and meteorology.**

Parameter	No.	Mean ± SD	Minimum	Maximum
Endothelial function parameters				
Brachial artery ultrasound				
Brachial artery diameter at baseline (mm)^a^	84	3.96 ± 0.65	2.91	5.98
FMD (%)^a^	83	5.9 ± 3.9	0.4	16.3
NTGMD (%)^a^	78	13.4 ± 7.4	3.8	32.1
Pulse waveform				
LAEI (mL/mmHg × 10)^b^	51	16.5 ± 3.6	12.4	22.4
SAEI (mL/mmHg × 100)^b^	47	4.2 ± 1.6	1.7	7.1
SVR (dyne sec/cm^5^)^b^	51	1415.0 ± 244.0	959.6	1702.5
Network PM_2.5_				
PM_2.5_ (μg/m3)	383	13.6 ± 7.0	2.0	38.9
HSF rooftop meteorology				
Air temperature (°C)	385	15.9 ± 8.5	−6.5	31.5
Relative humidity (%)	385	62.5 ± 16.6	25.1	97.7
Barometric pressure (hPa)	386	1001.2 ± 6.5	981.4	1021.9

a Brachial artery ultrasound values are the mean from the patient mean of four measurements from 22 subjects

b Pulse waveform values are the mean from the patient mean of four measurements of 13 patients; one patient was excluded for the SAEI because of extremely high values.

**Table 4:** **Percent change of mean endothelial function and vascular compliance parameters per 10-μg/m**^3^ **increase in PM2.5** **[estimate (95% CI)].**

PM_2.5_ days before clinical measurement	FMD	NTGMD	LAEI	SAEI	SVR
Lag 0	−17.3 (−34.6 to 0.0)*	2.5 (−9.0 to 13.9)	0.4 (−4.2 to 5.0)	−3.0 (−13.0 to 7.0)	−1.6 (−3.7 to 0.4)
Lag 1	−4.4 (−24.6 to 15.8)	−13.6 (−24.5 to −2.6)*	−0.3 (−6.0 to 5.4)	−17.0 (−27.5 to −6.4)**	1.6 (−0.9 to 4.1)
Lag 2	−18.6 (−44.8 to 7.6)	−10.2 (−23.5 to 3.0)	2.5 (−4.3 to 9.4)	−9.7 (−23.5 to 4.2)	3.5 (0.5 to 6.5)*
Lag 3	1.6 (−23.6 to 26.9)	−8.0 (−22.4 to 6.4)	−7.3 (−13.5 to −1.1)*	−15.1 (−29.3 to −0.9)*	2.4 (−0.5 to 5.3)
Lag 4	18.4 (−3.5 to 40.3)	3.6 (−7.9 to 15.0)	−2.3 (−8.0 to 3.3)	−2.1 (−14.0 to 9.7)	3.2 (0.7 to 5.6)*
Five-day average	−19.4 (−62.6 to 23.8)	−19.4 (−44.3 to 5.5)	−4.6 (−15.3 to 6.1)	−25.4 (−45.4 to −5.3)*	4.5 (−0.3 to 9.2)

* *p* < 0.05.

** *p* < 0.01.
